# Comparative Life Cycle Assessment (LCA) of Porous Asphalt Mixtures with Sustainable and Recycled Materials: A Cradle-to-Gate Approach

**DOI:** 10.3390/ma16196540

**Published:** 2023-10-03

**Authors:** Beatrice De Pascale, Piergiorgio Tataranni, Alessandra Bonoli, Claudio Lantieri

**Affiliations:** Department of Civil, Chemical, Environmental and Materials Engineering, University of Bologna, 40131 Bologna, Italy; piergiorg.tataranni2@unibo.it (P.T.); claudio.lantieri2@unibo.it (C.L.)

**Keywords:** life cycle assessment, cradle-to-gate, porous asphalt, CDW aggregates, polyolefin-based synthetic transparent binder

## Abstract

The road and construction sectors consume a large number of natural resources and energy, contributing significantly to waste generation and greenhouse gas emissions (GHG). The use of recycled aggregate from construction and demolition waste as a substitute for virgin aggregate is a current practice in the construction of new road sections. Additionally, in recent years, there has been an increasing focus on finding alternatives to bitumen for binders used in asphalt mixes. This study investigates and compares the impacts associated with two porous asphalt mixtures produced with CDW aggregates, virgin aggregates, and a polyolefin-based synthetic transparent binder through an LCA methodology. A cradle-to-gate approach was employed. Model characterization for calculating the potential environmental impacts of each porous asphalt mixture was performed using the ReCipe 2016 assessment method at the midpoint and endpoint levels. The results are presented with reference to a baseline scenario corresponding to a porous asphalt mixture, confirming the benefits associated with the use of recycled aggregates and in some cases the benefits of not using bitumen-based binders. This work contributes to the understanding of the importance of choosing the least environmentally damaging solution during the production or rehabilitation of road pavement infrastructure.

## 1. Introduction

The infrastructure field is one of the most relevant sectors of the European economy but at the same time one of the most detrimental to nature and to the surrounding environment due to its activity [1]. Almost 40% of raw materials extracted from the lithosphere are consumed in the road and construction sectors [2]. That corresponds approximately to 50% of global greenhouse emissions [3,4]. It is well known that primary raw materials used in road pavement production are scarce and their exploitation demands an urgent solution [5]. Being aware of the important role that the infrastructure sector represents in modern society, the European Commission set (in the Transport white paper) the target of reducing the emission associated with it by 60% by 2050 [6]. Furthermore, in Agenda 2030, one of the goals is to enhance the reuse and recycling of waste generated and avoid landfilling disposal [7]. A sustainable option to be adopted is to use recycled materials [8]. In order to achieve this target in the road sector, pavement engineers and researchers are studying different technologies to incorporate waste products coming from different sectors in the production of more eco-friendly pavement materials. Different types of recycled materials can be used in the production of road pavements and the use of construction and demolition waste (CDW) is widely spread to produce based and sub-base layers of pavements [9,10]. CDW is a heterogeneous waste material composed of several different constituents, outlined in Chapter 17 of the European Waste Catalogue [11]. One of the main components of construction and demolition waste is recycled concrete aggregate (RCA). With more than 10 billion tons per year produced all over the world, RCAs are solid wastes with higher recycling potential [12,13]. They result from the crushing of concrete from buildings and from the rigid pavement at the end of their life [14]. If previously investigated and properly qualified, RCAs can be used in the surface layer of road pavements and the always-incrementing use of them can lead to fewer tons of materials being taken into landfill every year [15]. Having said that, using RCAs as aggregates inside a surface layer of pavements could create an increment in environmental benefits in urban areas. A precise and specific regulation for the use of recycled aggregates in road construction and sector could lead to an increment in the recycling rate for all type of roads and road layers [16].

It has been widely proved that the majority of the environmental burdens and the most energy-demanding process in asphalt pavement construction is bitumen production [17,18,19]. It is demonstrated that bitumen has the highest impact in terms of global warming potential compared to raw materials due to the crude oil extraction and production process to create it [20]. Furthermore, the production phase of asphalt mixture accounts for more than 50% of the total emission related to the life cycle of road pavements, with bitumen as the leading contributor [21]. The research is advocating for the use of a non-bituminous binder, and in the context of urban pavements and UHI mitigation, light-coloured binders represent the right solution [22]. Usually, coloured and cool pavements are produced with the use of a bitumen-based clear binder, coloured pigments, or transparent resin [23]. An innovative solution is analysed in this paper, where the environmental burdens of a porous asphalt mixture made with a polyolefin-based synthetic transparent binder are investigated. Different studies already highlighted the thermal performances of a resin-based binder used for the surface layer and evaluated its environmental performances [24,25]. Furthermore, the use of RCAs together with these transparent binders contribute to creating “cool pavement”, which is a technology used for reducing the absorption of heat and resulting in a lower surface temperature [26]. These aspects are linked to the UHI phenomenon and are significantly influenced by the pavement developed during the recent few decades.

In this context of mitigation of the UHI phenomenon and reduction of the environmental impact associated with road pavement, porous asphalts are widely preferred [27,28]. Thanks to their properties of sound absorption and the capability of making the water infiltrate and refurbish the groundwater basins, permeable and porous pavements are reasonable solutions for reaching the European target. Moreover, a key process in the life cycle of road pavement is the transport of the materials and products associated with different phases, starting from the transport of raw materials to the transport of the waste to the landfills. As a result, a lot of studies underline that the bulk of emissions is associated with the transport process in different phases of the road life cycle [29,30]. Within this context, the most common way to evaluate the life cycle of a product, process, or activity is the life cycle assessment analysis. The life cycle assessment (LCA) is a well-known methodology used to evaluate the environmental performances associated with all stages of the life cycle of a product or process, based on the following international standards: ISO 14040 and ISO 14044 [31,32]. The LCA is not only crucial for assessing impacts on the environment from one stage of the life cycle to another, but it also helps decision-makers to compare multiple solutions and select the one less detrimental to the environment. Different studies have been carried out to assess the environmental impact of innovative road materials, enhancing the effects of using different wastes for a more sustainable asphalt mixture [33,34]. Here, comes the novelty of this research as it focuses mainly on the evaluation of the environmental impact of a porous asphalt mixture for urban pavements made with 50% of recycled concrete aggregates and a polyolefin-based synthetic transparent binder. The environmental burdens of the aforementioned materials are compared with a porous asphalt mixture composed of virgin aggregates and the polyolefin-based synthetic transparent binder to enhance the advantages connected to the use of recycled materials in new roads construction. For clarity of the data and the result, the environmental impacts of the two porous asphalt mixtures are normalised with respect to conventional porous asphalt, produced with a bituminous binder and virgin aggregates. The results show promising value for most of the indicators investigated in this research.

## 2. Materials and Methodology

### 2.1. Materials

**Figure 1:**
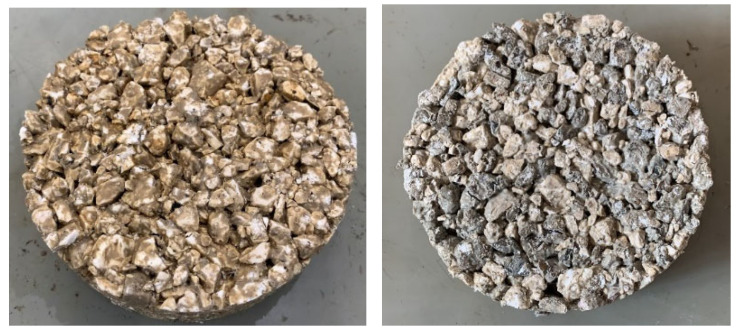
Application of the innovative binder [36].

Asphalt mixtures are usually composed of 95% of virgin aggregates and 5% of binder (usually, bitumen) that glue together the aggregates creating a cohesive mix [35]. This study aims to investigate and compare the environmental performances of two porous asphalt mixtures composed of virgin aggregates, recycled concrete aggregates, and an innovative polyolefin-based synthetic transparent binder. The two scenarios are defined and compared with a baseline scenario consisting of virgin aggregates and a polymer-modified bitumen, labelled as baseline_PA, which is traditionally used for porous asphalt. The virgin aggregates are pale limestone aggregates mainly composed of calcium carbonate coming from the province of Ancona, Italy. These specific aggregates are chosen since the innovative polyolefin-based synthetic transparent binder has the property of being transparent; hence, the light-coloured aggregates are needed to have a light-coloured pavement surface. Figure 1 represents an application of the innovative binder, taken from the producer archive.

The recycled aggregates used are construction and demolition waste aggregates, hereafter referred to as CDW aggregates, coming from the province of Bologna. The literature has shown a generic indication of CDW aggregates use in road pavement layers, according to which the main constituent of the materials should possibly be a crushed concrete and lytic material [37,38]. The CDW aggregates used in this study were previously analysed through laboratory investigation and they are composed of almost 90% of concrete products. The bitumen used is a polymer-modified bitumen 45/80–70 with SBS, specific for the use in porous asphalt. For completeness of the data, 0.3% of cellulose fibre was used as a stabilizing agent in the baseline_PA mixture to prevent bitumen from leaking. The polyolefin-based synthetic transparent binder is a commercial product, manufactured with polymers, resin, and oils. This binder is produced in Italy and has the characteristic to melt when it makes contact with the preheated aggregates at 170–180 °C. It is important to underline this property because no additional energy and heat are needed in the mixture production process to maintain the viscosity of the binder, which is necessary for the baseline bituminous scenario in order to allow the blending of bitumen and aggregates. The two different mixtures analysed in terms of environmental performance and the baseline scenario are listed and labelled as follows:

- Mix-I: totally composed of limestone virgin aggregates and the polyolefin-based synthetic transparent binder.
- Mix-II: composed of 50% of CDW aggregates, 50% of limestone virgin aggregates, and the polyolefin-based synthetic transparent binder.
- Baseline_PA: composed of limestone virgin aggregates and polymer-modified bitumen.

It is worth underlining that each mixture was previously tested in the laboratory to assess its functional and mechanical properties. The tests validated the experimental mix designs, confirming the suitability of the proposed mixtures to produce porous pavements [36].

### 2.2. Methodology

A life cycle assessment (LCA) analysis was carried out to evaluate and compare the environmental performances of the two porous mixtures. The LCA allows the estimation of the environmental impact of products, processes, or activities through the identification and quantification of energy flows and materials [39]. The management of the environmental impacts of a life cycle starts from the extraction of raw material to the production phase, construction, service life, and disposal [40,41]. In this study, the LCA was limited to the production phase, without the impact concerning the construction and service life phases of road pavement being investigated. The criteria given by the Standard-ISO 14040 and ISO 14044 describe the LCA procedure and encompass four defined phases of the analysis as follows: goal and scope definition, life cycle inventory, life cycle impact assessment, and life cycle interpretation analysis.

#### 2.2.1. Goal and Scope Definition

**Figure 2:**
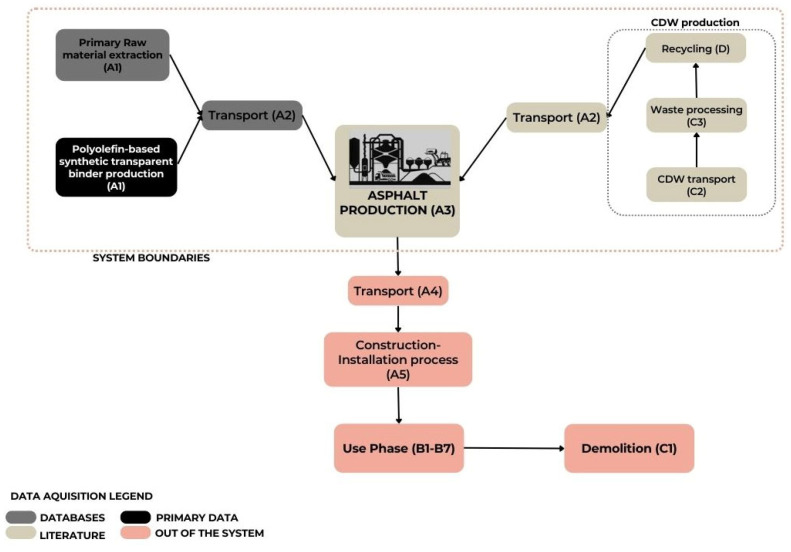
LCA system boundaries of this study with reference to the life cycle stages reported in EN 15804 (EN 15804, 2021 [44]).

The scope of this study is to assess and compare two porous mixtures made with 50% of CDW aggregates, virgin aggregates, and an innovative polyolefin-based synthetic transparent binder. A comparative LCA approach is adopted in order to emphasise the advantages of using CDW aggregates and a bitumen-free binder in asphalt concrete. The environmental impacts of the mixtures are determined and quantified by referring to specific environmental indicators reported in Table 1 and Table 2. These indicators are chosen to enable a complete assessment of the environmental impact connected to porous asphalt production of the two mixtures investigated. A cradle-to-gate approach is developed for the LCA analysis. As shown in Figure 2, the system boundaries include the (1) extraction of primary raw materials and the production of the polyolefin-based synthetic transparent binder; (2) CDW aggregates production, involving the transport of the construction and demolition waste from the production site, the waste processing, and the recycling process; (3) transportation of primary raw materials and CDW aggregates to the asphalt plant; (4) production of the porous asphalt mixtures to the plant.

**Table 1:** Life cycle inventory data for 1 kg of Mix-II production.

Data Set	Data Source	Unit	Data Value
CDW production	[42]	kg	0.473
Polyolefin-based synthetic transparent binder	Chemical Company	kg	0.054
Gravel, crushed (GLO)|market for|	Ecoinvent v.3.7	kg	0.4257
Limestone, crushed, for mill (GLO)|market for|	Ecoinvent v.3.7	kg	0.0473
Production phase	[43]	p	1

All the processes involving the construction phase, use phase, demolition phase, and landfill or recycling operation are excluded from this study. The declared unit (DU) is defined in relation to the aim of this study and in accordance with EN 15804, and it is equal to 1 kg of manufactured porous asphalt mixture [44]. As already mentioned, the two porous asphalt mixtures are defined and compared with a baseline scenario consisting of 94.3% of the same virgin aggregates, 0.3% of cellulose fibre, and 5.4% of polymer-modified bitumen. For modelling and evaluating the life cycle impacts the SimaPro 9.2 software is used. In order to ensure a representative production process, the inventory data are both primary data, specifically for the polyolefin-based synthetic transparent binder, and secondary data coming from Econinvent v.3.7 databases and the USLCI database. The impact assessment chosen is the ReCipe 2016 both at midpoint and endpoint levels to ensure a comprehensive assessment of the potential environmental burdens connected to the use of the polyolefin-based synthetic transparent binder and the CDW aggregates in new pavement construction.

#### 2.2.2. Life Cycle Inventory

The LCI for each process of the analysed asphalt mixtures is a combination of primary and secondary data. The data relating to the polyolefin-based synthetic transparent binder were obtained by the chemical company, which is the exclusive producer of the binder. The exact percentage of the constituent materials is not publishable due to patent reasons. Thanks to the information collected from the company in terms of materials, transport, and energy, it was possible to have representative data and values for performing this study. All the other processes were taken from secondary data. The virgin aggregates required to produce the porous mixtures were designed as gravel and the LCI data connected to this were taken from the unit process “gravel, crushed (GLO)|market for gravel, crushed” of the Ecoinvent v.3.7 database. The same database was used for designing the process connected to the filler and to the cellulose fibre that was involved in the baseline_PA process (“limestone, crushed for mill (GLO)|market for limestone, crushed, for mill”; “cellulose fibre, inclusive blowing in (GLO)|market for”. The LCI data corresponding to the process of polymer-modified bitumen binder were obtained using the Eurobitume manual to produce the SBS as well as with the unit process “Bitumen, at refinery” from the database USLCI for the neat bitumen [45]. As a result, the final production of polymer-modified bitumen was developed in accordance with the aforementioned manual. To define the process of the CDW aggregates, data concerning the construction and demolition waste management and production were collected from the literature [42] Having no primary data, which were geolocalised in Italy, the production process connected to the production of the porous asphalt mixture was taken from the life cycle inventories found in the literature [30,45]. As already mentioned, the production process of the two porous mixtures developed with the polyolefin-based synthetic transparent binder was designed without considering the energy needed to keep the binder viscous, since the innovative material melts only when in contact with the hot aggregates. During the assembly of the “Production phase”, the unit process connected to the heat for maintaining the bitumen fluid was not considered. A summary of the LCI to produce 1 kg of porous asphalt mixture containing the CDW aggregates and the polyolefin-based synthetic transparent binder is shown in Table 1.

#### 2.2.3. Life Cycle Impact Assessment

The next step of the LCA methodology is performing the assessment of the life cycle impacts. SimaPro 9.2 is used for LCA modelling and the development of the analysis. The inventories are analysed using the ReCipe 2016 midpoint and endpoint methods. The midpoint method includes 18 midpoint impact categories expressed in a reference substance unit, as well as the endpoint level including 3 different damage assessments as follows: human health, ecosystem, and resource availability. The 18 midpoint or problem-oriented indicator of the proposed method are characterized and explained as follows:

- Climate change, in terms of global warming potential, based on the IPCC 2013 report and expressed in kg CO_2_ equivalents.
- Ozone depletion accounts for the disruption of the stratospheric ozone layer by anthropogenic emissions of an ozone depleting substance. Expressed in kg of CFC-11 equivalents [46].
- Ionizing radiation accounts for the level of exposure for the global population, in terms of kBq Cobalt-60 equivalents in air.
- Fine particulate matter formation, expressed as the intake fraction of PM2.5, with kg of PM2.5 equivalents as units of measure.
- Photochemical ozone formation, in terms of human health and terrestrial ecosystem, characterized by the intake rate of ozone due to change in the emission. The unit for both of them is kg/NO_x_ equivalents.
- Terrestrial acidification, accounts for the acidification potential (AP) derived using the emission weighted world average fate factor of SO_2_ [46].
- Freshwater eutrophication, accounts for the environmental emission of P containing nutrients and expressed in kg of P to freshwater equivalents.
- Marine eutrophication, accounts for the environmental emission of N containing nutrients and expressed in kg of N to marine equivalents.
- Human toxicity and ecotoxicity, accounts for the environmental persistence and accumulation in the human food chain, and the toxicity of a chemical. They are expressed in terms of kg 1,4-dichlorobenzeen (1,4 DCB) emitted.
- Land use and water use, expressed in m^2^ per year and in m^3^ of water consumed, respectively.
- Mineral resource scarcity and fossil resource scarcity, expressed in kg of copper (Cu) equivalents for the first one and in kg oil equivalents for the second one.

A fulfilling list of the environmental categories is reported in Table 1 and Table 2. All the results are reported based on the baseline scenario and the indicators are selected to enable a complete and comprehensive assessment of the potential environmental damages connected to the use of the analysed porous asphalt mixtures.

## 3. Results and Discussion

**Figure 3:**
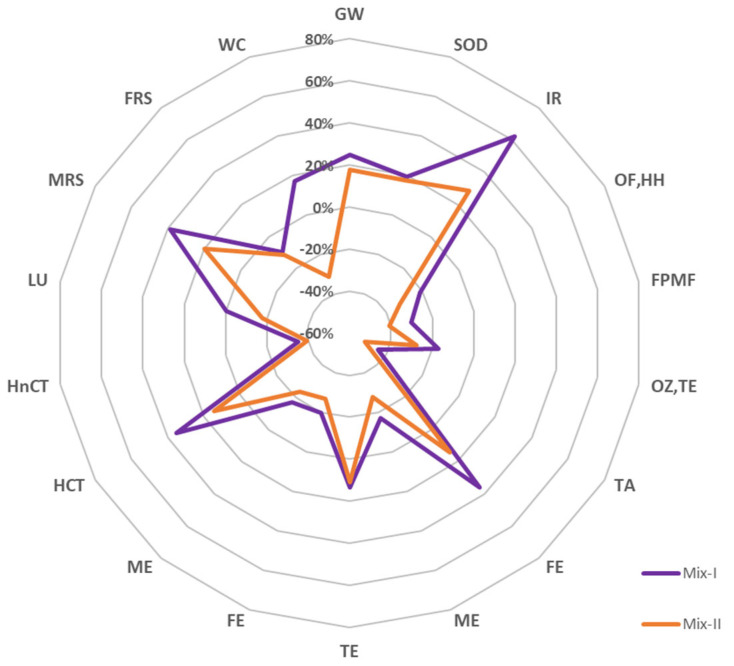
Relative variations of mid−point results with respect to the baseline scenario. **Acronyms**: GW: global warming; SOD: stratospheric ozone depletion; IR: ionizing radiation; OF, HH: ozone formation, human health; FPMF: fine particulate matter formation; OF, TE: ozone formation, terrestrial ecosystem; TA: terrestrial acidification; FE: freshwater eutrophication; ME: marine eutrophication; TE: terrestrial ecotoxicity; FE: freshwater ecotoxicity; ME: marine ecotoxicity; HCT: human carcinogenic toxicity; HnCT: human non−carcinogenic toxicity; LU: land use; MRS: mineral resource scarcity; FRS: fossil resource scarcity; WC: water consumption.

**Figure 4:**
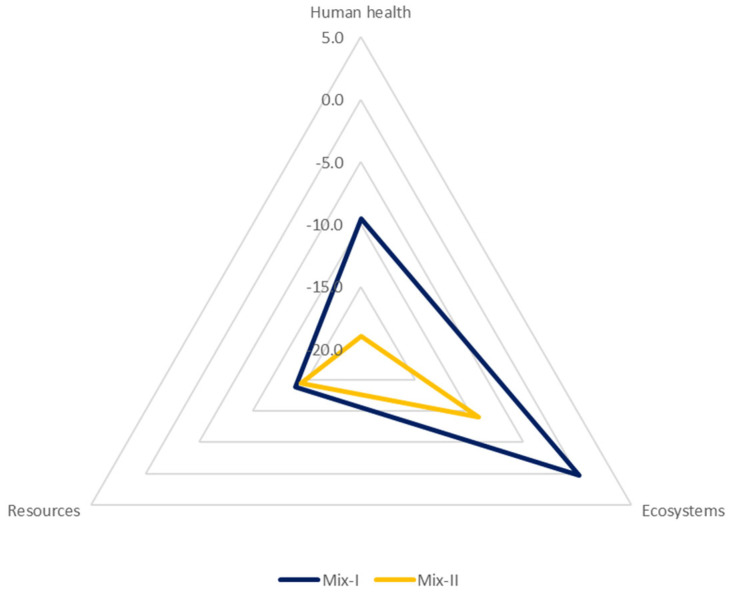
Relative variation of end−point results with respect to the baseline scenario.

The prime objective of the LCA analysis was to investigate the environmental performances of a porous asphalt mixture made with a polyolefin-based synthetic transparent binder and 50% of CDW aggregates and compare it with a mixture produced with virgin aggregates. For this study, the declared unit defined is 1 kg of a porous mixture and is in compliance with the European standard EN-15804. The results of the analysis are reported and compared with a baseline scenario, named baseline_PA, to assess and show the benefit connected with the innovative mixtures. Table 2 and Table 3 show the results for the two mixtures and the baseline scenario. The relative variations of LCA results of the porous mixture to the baseline_PA are reported in Figure 3 and Figure 4 for the midpoint level and endpoint level, respectively.

**Figure 5:**
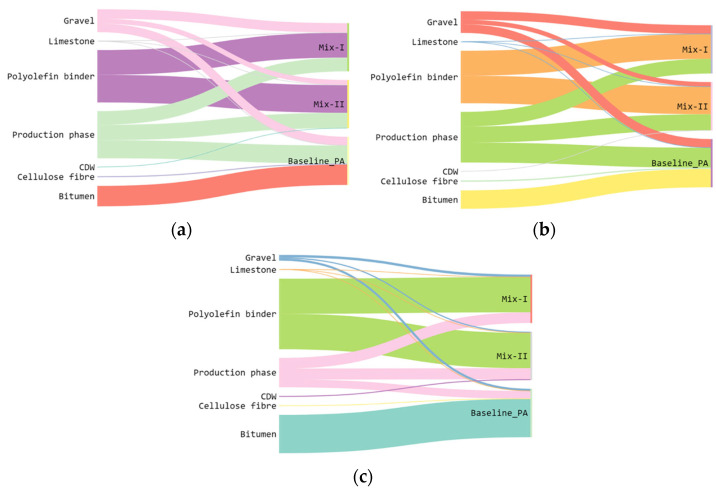
Sankey diagram for the sub-process contribution of Mix-I, Mix-II, and baseline scenario to the damage categories of (**a**) human health, (**b**) the ecosystem, and (**c**) resources.

As other studies have already proved, the most environmentally detrimental process is the one connected to the bitumen [47,48,49]. Looking at the results of this study, it is possible to see that at midpoint level, Mix-I, which is composed of virgin aggregates, has a higher impact with respect to the baseline scenario for at least 50% of the impact categories analysed. This is mainly due to the composition of the polyolefin-based synthetic transparent binder. The innovative binder is not bitumen-based but some of its constituents are polymers and oils that are fossil fuel-based. This leads to high impact, especially looking at the indicators such as global warming, stratospheric ozone depletion, and ionizing radiation. Furthermore, almost 50% of its constituent materials undertake a long journey before arriving at the production plant and this highly affects the impact connected to the production of the binder. All these statements are well represented in Figure 5, where the contributions at the endpoint level of the polyolefin-based synthetic transparent binder and of the bitumen are evident. The Sankey diagrams reported in Figure 5 represent the contribution of each mixture’s constituents for the three damage categories analysed. On the other hand, thanks to the use of CDW aggregates, it is possible to view important changes in the analysis. In all the endpoint values, the mixture containing the CDW aggregates observe lower results if compared with the other mixtures, which is in line with the findings of other studies [35,50]. It is certain that all the midpoint categories show results lower than their virgin counterpart. However, some categories are still higher than the baseline scenario, and this is associated with the production process of the polyolefin-based synthetic transparent binder.

By looking at the baseline_PA scenario, bitumen certainly remains the most impactful material within the production process of asphalt concrete but, as this study shows, replacing bitumen with other binders that are still fossil fuel-based does not bring substantial benefits. Nonetheless, the benefits connected to the use of recycled aggregates rather than virgin aggregates are evident. As can be seen from the results above, despite the presence of the polyolefin-based synthetic transparent binder, it is possible to achieve significant environmental benefits and reduce the impact associated with the production of asphalt concrete with the use of CDW aggregates.

## 4. Conclusions

This study analysed the environmental potential of a porous mixture made of recycled concrete aggregates and a polyolefin-based synthetic transparent binder and compared it with a mixture made with virgin aggregates and the same innovative binder. The LCA was performed in compliance with the ISO 14040 series. ReCipe 2016 impact assessment method was adopted to investigate the environmental performances of the two different porous asphalt mixture scenarios at the midpoint and endpoint levels. The main conclusion and findings of this research are as follows:

- The use of the polyolefin-based synthetic transparent binder increases the environmental burden in the impact categories connected to global warming potential.
- Despite the fact that the polyolefin-based synthetic transparent binder is mainly composed of polymers, resin, and oil, and not bitumen, the high impact is associated with its virgin constituents, most of which are fossil fuel-based.
- The transport and production of the polyolefin-based synthetic transparent binder components play a key role in the impact assessment. Through the LCA of the innovative binder, it was possible to see that the transport of its constituents affects its environmental performance.
- CDW aggregates benefits, when compared with the baseline scenario, are not so evident for the categories concerning the global warming potential, since the bulk of the emission associated with it is connected to the production of the innovative binder.
- Nonetheless, it is possible to state that the use of CDW aggregates is beneficial since all the environmental impacts associated with Mix-II are lower compared to its virgin counterpart.
- However, as stated by different studies, possible additional environmental impacts of CDW aggregates are associated with their management; hence, the CED indicator should be considered in future studies [51,52].
- Mix-II is the most environmentally friendly porous asphalt mixture with respect to the impact categories of mineral resource scarcity, land use, and water consumption.

The use of recycled materials in the design and construction of new roads, especially in an urban area, is of great importance [19,53]. From this research, it is possible to state that using a different type of binder, classified as no-bituminous, does not necessarily lead to a decrease in the impact associated with the production of an asphalt mixture. A life cycle assessment considering a cradle-to-cradle approach should be investigated in a future study to examine the environmental potential of the analysed mixtures in their whole life cycle. Future studies should focus on the use phase of the innovative pavement since its beneficial contribution is mostly attributed to its transparent and light colour. During the use phase the innovative binder would contribute positively to the decrease of the UHI connected to the road pavements. Hence, the use of this binder does not affect the useful life of a road pavement since the mechanical performance of it is totally comparable with that of traditional bituminous porous asphalt. Furthermore, a sensitivity analysis to produce the polyolefin-based synthetic transparent binder should be developed to understand the most detrimental process connected with it.

## Figures and Tables

**Table 2:** LCIA results for the two porous asphalt mixtures and the baseline scenario at the midpoint level.

Impact Category	Unit	Type of Mixtures		
		Baseline_PA	Mix-I	Mix-II
Global warming	kg CO2 eq	0.09248	0.11543	0.10904
Stratospheric ozone depletion	kg CFC-11 eq	3.25 × 10^−8^	3.87 × 10^−8^	3.81 × 10^−8^
Ionizing radiation	kBq Co-60 eq	0.00205	0.00333	0.00264
Ozone formation, human health	kg NOxeq	0.00039	0.00031	0.00027
Fine particulate matter formation	kg PM2.5 eq	0.00022	0.00015	0.00013
Ozone formation, terrestrial ecosystem	kg NOxeq	0.00042	0.00035	0.00030
Terrestrial acidification	kg SO2 eq	0.00064	0.00040	0.00031
Freshwater eutrophication	kg P eq	1.60 × 10^−5^	2.18 × 10^−5^	1.83 × 10^−5^
Marine eutrophication	kg N eq	2.06 × 10^−6^	1.71 × 10^−6^	1.50 × 10^−6^
Terrestrial ecotoxicity	kg 1.4-DCB	0.40715	0.46158	0.45212
Freshwater ecotoxicity	kg 1.4-DCB	0.00243	0.00194	0.00178
Marine ecotoxicity	kg 1.4-DCB	0.00350	0.00291	0.00268
Human carcinogenic toxicity	kg 1.4-DCB	0.00258	0.00349	0.00296
Human non-carcinogenic toxicity	kg 1.4-DCB	0.09591	0.06229	0.05817
Land use	m2a crop eq	0.00243	0.00242	0.00199
Mineral resource scarcity	kg Cu eq	0.00017	0.00024	0.00020
Fossil resource scarcity	kg oil eq	0.08427	0.07588	0.07449
Water consumption	m3	0.00111	0.00129	0.00076

**Table 3:** LCIA results for the two porous asphalt mixtures and the baseline scenario at the endpoint level.

Damage Category	Unit	Type of Mixtures		
		Baseline_PA	Mix-I	Mix-II
Human Health	DALY	2.56 × 10^−7^	2.31 × 10^−7^	2.07 × 10^−7^
Ecosystem	species.yr	5.02 × 10^−10^	5.03 × 10^−10^	4.56 × 10^−10^
Resources	USD 2013	0.035221	0.030331	0.030125

## Data Availability

Other data are not available due to confidentiality.
